# African swine fever; insights into genomic aspects, reservoirs and transmission patterns of virus

**DOI:** 10.3389/fvets.2024.1413237

**Published:** 2024-08-13

**Authors:** Bader S. Alotaibi, Chia-Hung Wu, Majid Khan, Mohsin Nawaz, Chien-Chin Chen, Abid Ali

**Affiliations:** ^1^Department of Clinical Laboratory Sciences, College of Applied Medical Sciences, Shaqra University, Riyadh, Saudi Arabia; ^2^Division of General Surgery, Department of Surgery, Ditmanson Medical Foundation Chia-Yi Christian Hospital, Chiayi, Taiwan; ^3^Department of Zoology, Abdul Wali Khan University Mardan, Mardan, Pakistan; ^4^Faculty of Veterinary and Animal Sciences, University of Poonch Rawalakot Azad Kashmir, Rawalakot, Pakistan; ^5^Department of Pathology, Ditmanson Medical Foundation Chia-Yi Christian Hospital, Chiayi, Taiwan; ^6^Department of Cosmetic Science, Chia Nan University of Pharmacy and Science, Tainan, Taiwan; ^7^Ph.D. Program in Translational Medicine and Rong Hsing Translational Medicine Research Center, National Chung Hsing University, Taichung, Taiwan; ^8^Department of Biotechnology and Bioindustry Sciences, College of Bioscience and Biotechnology, National Cheng Kung University, Tainan, Taiwan

**Keywords:** African swine fever virus, pigs, ticks, recombinant vaccines, *Ornithodoros*

## Abstract

African swine fever is a hemorrhagic disease of pigs with high mortality rates. Since its first characterization in 1921, there has been sufficient information about African swine fever virus (ASFV) and related diseases. The virus has been found and maintained in the sylvatic cycle involving ticks and domestic and wild boars in affected regions. The ASFV is spread through direct and indirect contact with infected pigs, their products and carrier vectors especially *Ornithodoros* ticks. Severe economic losses and a decline in pig production have been observed in ASFV affected countries, particularly in sub-Saharan Africa and Europe. At the end of 2018, the ASFV adversely affected China, the world’s leading pork-producer. Control strategies for the disease remained challenging due to the unavailability of effective vaccines and the lack of successful therapeutic measures. However, considerable efforts have been made in recent years to understand the biology of the virus, surveillance and effective control measures. This review emphasizes and summarizes the current state of information regarding the knowledge of etiology, epidemiology, transmission, and vaccine-based control measures against ASFV.

## Introduction

African swine fever virus (ASFV) belongs to the family Asfarviridae is a double-stranded DNA virus that causes African swine fever in Suidae (1, 2). In its ancestral African habitat, the ASFV evolved approximately 300 years ago in its arthropod vector in a sylvatic cycle, specifically involving common warthogs (*Phacochoerus africanus*) and the soft tick (*Ornithodoros moubata*) (3). The ASFV is epidemic in nature, causing large-scale mortality in the infected pig population (4). Serious economic consequences accompany outbreaks of the disease and therefore require proactive surveillance and management (5). Horizontal transmission of ASFV occurs through the feeding of swill containing infectious pig meat, contaminated pig-related products, and competent vector species, especially *Ornithodoros* ticks (6). Transmission of the ASFV occurs within warthog burrows, primarily between ticks and warthogs (7).

In the late 1800s, the swine industry became larger in Kenya under British colonization, which significantly contributed to the prevalence of ASF and its subsequent distribution in Africa (8). The virus spread out of Africa in two different instance. The first incursion occurred in 1957 in Portugal, where ASFV-infected and contaminated waste materials were thrown from airline to feed the pigs, leading to the virus spreading through Russia, Western Europe, the Caribbean and Brazil over three decades. However, except for Sardinia, the disease was eradicated from all these affected areas by the mid-1990s (9). In 2007, a second incursion occurred in Georgia, expanded to the Russian Federation and Eastern Europe, and then spread globally (3). Globally, China is the largest pork producing country that is adversely affected by ASFV. In Europe, successful precautionary measures were limited to the Czech Republic, Belgium, Sweden, and Germany. There are high risks of the ASFV introduction to the United States, which is the third largest pork producing country in the world after China and the European Union (10). In July 2021, the USDA confirmed the entry of ASF into the Dominican Republic which then reached Haiti by September, marking a considerable geographical invasion and emphasizing the risks of ASFV being introduced into mainland North America. Large-scale movements of humans and animals are the major threat to those countries, that are free from ASFV (11). The increase in global trade of various goods and animals within or outside the country, provides a favorable means in the transmission and expansion of ASFV (12). Reducing the risks of ASFV, it requires a global attention to limit further expansion of this havoc-transmitting agent. Comprehensive knowledge of ASFV is necessary to overcome and eradicate the disease; for this purpose, this review is designed to highlight the etiology, epidemiology, transmission sources, and current development in prevention and control measures against ASFV.

## Microbiology of ASFV

### Structure of the virus

**Figure 1:**
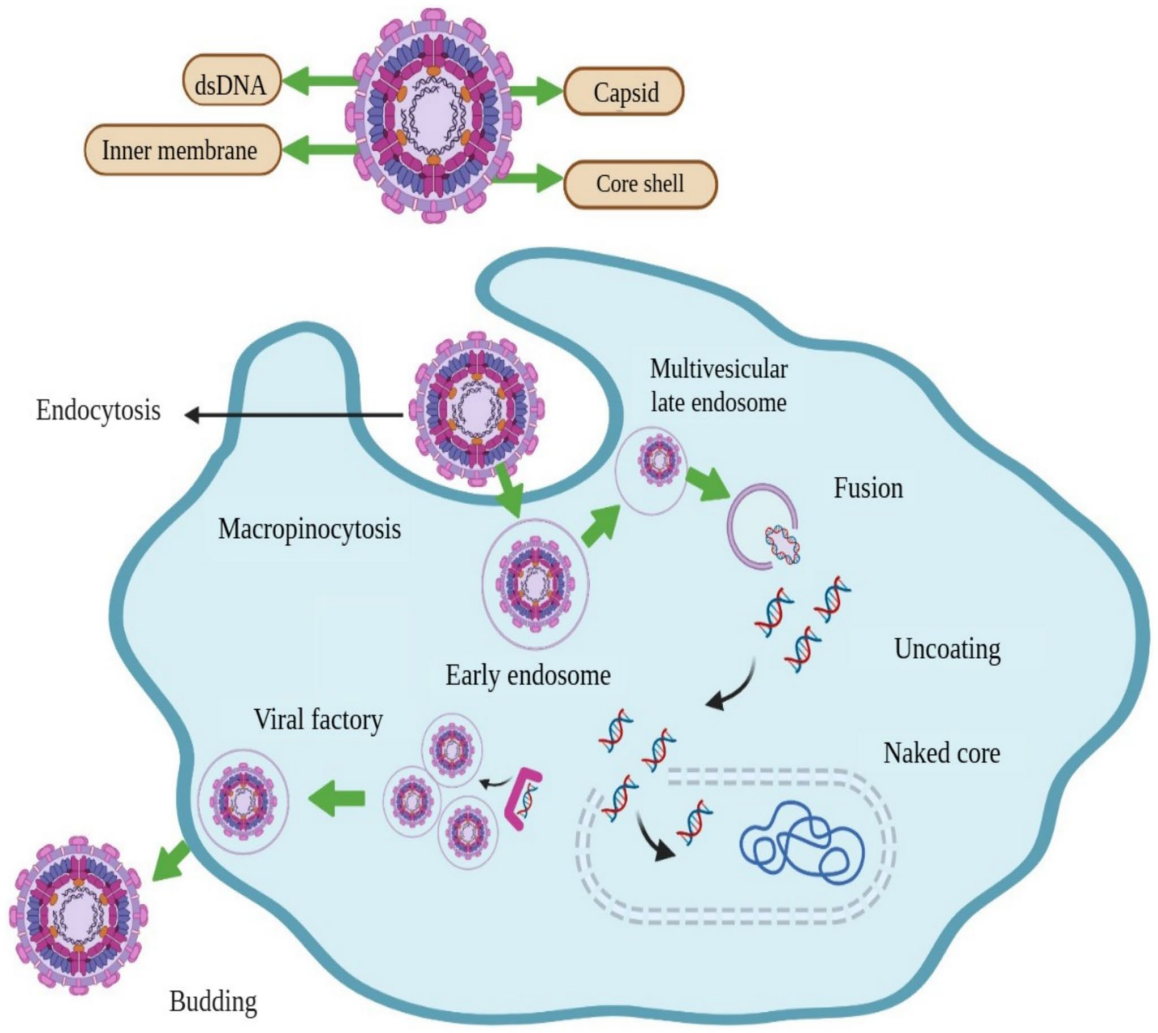
Structure and schematic presentation of African swine fever virus and mechanism of its entry into host cell, replication, and release.

The shape of ASFV is icosahedral, and has an average diameter of 200 nm. There are four concentric layers in the structure of ASFV, an outer hexagonal membrane acquired by originating through the cell plasma membrane. The capsid is the outermost layer of the virion. The internal core of the ASFV particle is formed by the central genome and contains nucleoids, which are coated by a thick capsid in a layer called the core-shell (Figure 1) (13). For immunological interactions with the host, viral genomes encode genes essential for the replication of virus assembly (1). The virus replication process occurs mainly in the cytoplasm of infected macrophages and monocytes; however, it has also been observed in the nucleus at the early stages of infection (14). The ASFV is enveloped by a two-membraned collapsed cisterna, which is originates from the endoplasmic reticulum (15). Four classes of viral genes have been identified comprising immediate-early, early, intermediate, and late transcripts. Viral genes are usually express before and after the onset of DNA replication (16, 17). Enzymes are required for the ASFV replication and are expressed after the entry of the virus into the cytoplasm (18).

### Genomic aspects

The ASFV genome is a single linear molecule of double stranded DNA having covalently closed double stranded DNA (19, 20). The P72 is the primary capsid protein, encoded by the *B646L* gene. The genotyping and sequencing of ASFV depend on a variable region of the *B646L* gene within the respective C-terminal of the gene. The ASFV genome ranges from 170 to 190 kilobases (kb) encoding 150–200 viral proteins, and has predicted open reading frames ranging from 151 to 186 kb (21). The ASFV has low mutation rate due to the accurate DNA proofreading.

The sequencing of the ASFV genome has resulted in the generation of several ASFV genomes of viral and low pathogenic isolates. Genomic analysis of ASFV has provided important information on structure, variation, and precise phylogenetic reconstruction (22). The *BA71V*, first strain of ASFV sequenced in 1995 (23), has been used as a comparative model for ASFV, which accumulates large scale information related to its biochemical, morphogenetic and genetic behavior (24). The genotyping and sequencing of ASFV depend on a variable region of the *B646L* gene within the respective C-terminal of the gene (25). Different virulent and pathogenic sequences have been identified and characterized from different origins and deposited in GenBank for information and experimentation (22). Until to 2018, 19 full-length sequences of ASFV were available which were generated using the Sanger sequencing; the number of sequences increased to 114 in October 2021 (27, 26). The multigene family (MGF) are responsible for the variation of ASFV genomes (1). The deletion and insertion of copied regions occur within these five MGF genes, suggesting the role of MGF in generating antigenic variability, thus helping the virus to evade host immune response (28). Based on the *B646L* gene, ASFV is categorized and divided into 24 different genotypes, the p72 capsid protein is coded by the *B646L* gene of ASFV (14).

Different isolates of ASFV can induce variable severity of infection. Although mild and moderate complications are caused by mutated strains (1). Pathogenic and virulent strains of ASFV are responsible for mild or severe infection accompanied by symptoms like hemorrhages in the skin and internal organs with a high fever and at the final stage causes death. Sudden deaths of animals can lead to 90% of fatalities (29). It has been suggested that the range of host and virulence of ASFV depend on the members of MGF 360 and 505. Some reports have demonstrated that the removal of eight genes from family 360 and two genes from family 505 affects its virulence to infect macrophages (30). The virulence of ASFV is due to four genes: the thymidine kinase coding genes, 9GL (B119L in BA71V), United Kingdom gene (DP96R in BA71V) and NL-S gene (DP71L in BA71V) (31). Therefore, further studies are necessary to understand the viral genome of ASFV as well as the genes associated with its pathogenicity: such genes will be supportive of the development for efficient diagnosis and treatment.

Genome sequences highlight variations between viruses in terms of insertions, deletions, or mutations. Comparative genomic analysis facilitates evolutionary studies. In this context, the first detected variation was the insertion of tandem repeat sequences (TRS), of the 10-nucleotide “TATATAGGAA” present between *173R* and *1,329*. This insertion has been recognized for the first time in ASFV infecting wild boar in Poland and Lithuania in 2014, and now it is considered a new sub-genotype marker (14).

In China, comparison of identified sequences of two strains, DB/LN/2018 and Pig/HLJ/2018 indicated mutations due to the insertion and deletion of nucleotides at multiple positions in the genome (32). The length of the genome sequence of both viruses was found to be 189,404 bp, very similar to the genomes of PoL/2017, GA/2007/ and ASFV-SY18. A comparison of DB/LN/2018 and Pig/HLJ/2018 with GA/2007 revealed 16 inserted nucleotides and 9 deleted nucleotides at 15 and 5 positions, respectively. In the PoL/2017 viruses three deletions appeared, whereas no insertion or deletion is present in ASFY-SY18. Furthermore, five mutations observed in the ORFs of Pig/HLJ/2018 and DB/LN/2018, ASFV-SY18 and PoL/2017 strain. However, these mutations were not observed in the GA/2007 genome. Due to mutations in ASFV, as a result of insertion and deletion of nucleotides, it is confirmed that changes and alterations occur in ORFs. Although the comparisons with the database provided clues about genes that may modulate the virus-host relationship. It remains to be examined how these alterations occur and affect the pathogenic properties of the ORFs of ASFV.

### Epidemiological profiles of ASFV

Warthogs and domestic pigs are the main reservoirs for the ASFV. It is known from the last 10 years’ data on ASF in the global context that the country will be potentially at risk where pigs are commercially as well as having wild reservoirs. Ticks are responsible for the transmission of ASFV and act as “reservoirs.” Infected animals blood has the highest concentration of ASFV; therefore, virus transmission occurs through direct and close interaction with infected animals. Use of infected pork products and fomites or contact with them and the mechanical vectors, e.g., biting flies, may also aid in the transmission of the ASFV to the hosts which are uninfected from ASFV (33).

The ASF is not zoonotic; there are only reports of animal infections and transmission; the epidemiology of ASF reflects the circulation of the virus within animal and arthropod reservoirs. The prevalence of the disease varies from region to region due to epidemiological differences. Virus epidemics or outbreak situations have been observed based on the geographical conditions of the associated area. Large-scale outbreaks of ASFV usually occur between 2007 (China, Thailand) and 2009 (Vietnam) (34).

### Reservoirs of the ASFV

Although pigs are the most frequent reservoirs of ASFV, they exist in the sylvatic cycle between arthropod vectors specifically soft ticks, *O. moubata* and wild Suidae especially warthogs (35). Warthog burrows are the habitat of these ticks, where virus transmission occurs between warthogs and ticks (2). Recently, ASFV has been shown to grow within leeches (36), suggesting the participation of these hosts in the environmental perseverance of the virus (37).

### Role of Suidae

Members of the pig family known to be susceptible to ASFV include domestic pigs and wild boars (*Sus scrofa*), bush pigs (*Potamochoerus larvatus* and *Potamochoerus porcus*), warthogs (*Phacochoerus* spp.) and giant forest hogs (*Hylochoerus* spp.) (7, 38. Since wild boars and domestic pigs are the main reservoirs responsible for disease outbreaks, the prevention and control of infected pig herds are the main issues (39). Wild boars and domestic pigs show various clinical signs, from acute to chronic (40). Although death is the first indication of the disease in a per-acute form, loss of appetite, depression, cutaneous hyperaemia and elevated body temperature (> 41°C) are other clinical manifestations associated with the per-acute form. The acute form of the disease is typically characterized by pulmonary edema, respiratory distress, abortion in pregnant females, nasal and conjunctival discharge, skin hemorrhages, splenomegaly, extensive necrosis and high mortalities (41). For the first time, the chronic form of the disease was reported in the Iberian Peninsula. The chronic form of ASFV has also been evidenced in experimental inoculation of animals with European isolates (14). Wild boar shows the same signs as domesticated pigs; however, no signs are observed before death in virulent strains. In warthogs and bush pigs, ASF is most frequently asymptomatic (42). It has been observed that survivors of ASFV may not only play a key role in ASFV perseverance in endemic regions but also contribute to periodic incidences, outbreaks, and invasions of ASFV to uninfected animals in the disease free zone (43).

### Role of ticks

Following the classical epidemiological patterns of infectious diseases, ASFV circulates between animals and blood sucking arthropods. Hematophagy has been considered as a critical factor for the transmission and acquisition of ASFV by arthropods. Like other vector-borne diseases, the presence of an arthropod and blood sucking vector is important for the invasion and transmission of ASFV from the reservoir to healthy animals (44). An important aspect of the epizootic cycle of ASFV is the specificity of the vector. Among ticks, soft ticks are known competent vectors for ASFV. This pathogen has been isolated from 20 species of soft ticks and many other hematophagous arthropods, including competent louse and flies (Table 1). Even leeches have been considered susceptible to ASFV. Moreover, leeches were also able to transfer ASFV to experimental animals (36). Viral DNA has also been detected in hard ticks (*Dermacentor reticulatus* and *Ixodes ricinus*) collected from the bodies of dead ASFV positive wild boars and also in flies (54).

**Table 1:** Arthropods in which African swine fever virus has been detected.

Tick species	References
*D. reticulatus* *I. ricinus* *A. americanum* *A. mixtum*	(45)
*O. porcinus*	(46)
*O. erraticus*	(47, 48)
*O. moubata*	(49)
*O. coriaceus* *O. parkeri* *O. tunicate* *O. puertoricensis*	(50)
Other arthropods *Muscadomestica* (House fly)	(36, 51)
*Hirudomedicinalis* (leech)	(51)
*Drosophila* spp. (Fruit fly)	(51)
*Culicidae* spp. (Mosquitoes) *Haematopinussuis* (Swine lice) *Stomoxyscalcitrans* (Stable fly)	(52, 53)

*Ornithodoros* ticks play a major role in the transmission of ASFV. All stages of their development can be easily infected with the virus, in the blood meal when the virus is taken from an infected pig. However, under experimental conditions, not all ticks that feed on infected pigs or artificial membranes become infected. For instance, in the case of the *Ornithodoros erraticus* infection model, an infection rate of ticks of 83.1% (pig-feeding ticks) and 53.4% (membrane-feeding ticks) infection rate of ticks has been observed (48).

### Localization within tick

Rock (55) performed the first experiment on the localization of ASFV in, *Ornithodoros porcinus.* Initial replication of ASFV was observed in hemocytes (types I and II), epithelium of the midget, phagocytic cell, connective tissue, salivary gland, coxal gland and reproductive tissue, which were the secondary sites of virus replication. Similarly, the highest viral titers were detected in salivary glands and reproductive tissue after 91 days of infection (28).

### Survival of ASFV in ticks

It is known that ASFV once infects the tick, is capable of remaining viable within the tick’s body for a long period between 23 and 239 days, depending the tick species (56). Much longer survival has also been reported: 3 years in *O. moubata*, 5 years in *O. erraticus* and 502 days in *Ornithodoros coriaceus* (29, 50, 57). The data suggest that without contact with the swine population, the ASFV can survive within the tick population, therefore, making an alarming source of reinfection.

### Transstadial and transovarial transmission

Ticks can transmit infectious agents, retained within their body, to their next stage (transstadial) or the next generation (transovarial transmission). Similarly, soft ticks are believed to transmit ASFV transstadially, as well as transovarially through sexual contact, and directly to susceptible animals (29, 58, 59). However, in the case of transstadial transmission, the transmission rate of ASFV has been found to decrease with each molt (60).

### Pig-to-pig transmission

Direct contact with infectious pigs has been established to be an effective mechanism of disease transmission. The domestic pig can transmit the virus through nasal fluid, secretion, and in excretion through urine. In a recent study, healthy pigs housed together with infected ones became infected after one to 9 days after exposure. Delayed infectivity of healthy pigs was also observed in those separated from infected ones (14). During the fights, environmental contamination occurs due to the shedding of the blood from the wounds of infected pigs or from fecal contamination by infected Suidae (38). The possibility of a “carrier” state persists in pigs as well as in other Suidae. In the Netherlands, carrier pigs recovered from an acute ASFV infection with the lower pathogenicity strain were found to transfer the disease by direct contact with the uninfected pig population. It is believed that the overcoming and disappearance of clinical signs and symptoms occur, and after a month the infected pigs shed the virus. However, the effect on transmission will depend on the survival and duration of the carrier status of the pig (61).

### Transmission through the ingestion of contaminated feed

Direct contact with the environment or infected pigs can introduce the virus into their bodies through mucous membranes. However, some animals are infected via ingestion of infected food. The presence of ASFV has been confirmed in pork products such as pig fat, skin, meat, and other pig products used for different purposes (62). Similarly, seed contamination and fresh grass containing wild boars secretions are the key risks and threat for the transmission (63). Therefore, these sources should also be considered to prevent transmission to naive pigs.

### Wild boar-to-pig transmission

For ASFV transmissions, recent research has confirmed that wild boars act as a susceptible host for the transmission to domestic pigs. In domestic pigs, ASFV can develop nonspecific signs and symptoms (63). Several fields studies have confirmed the spread of ASFV from wild boars to domestic pigs. In this context, a study conducted in Russia detected several cases of ASFV primarily in wild boars before being observed in domestic pigs, and the death of wild boars caused by ASF was observed in the vicinity of ASF-affected farms (64). Similarly, the housing of susceptible pigs with ASFV-infected wild boars became infectious after 6 to 12 days post-exposure (65). The European Commission tasked the European Food Safety Authority (EFSA) to study and review the evolutionary ability and tendency of matrices, including arable crops, vegetables, wood chips, sawdust, hay, straw and other related agents that are the threats of transmitting ASFV (66).

Although ASFV is likely to transmit from wild boar to domestic pigs, long-range invasions of ASFV are mostly caused by anthropogenic activities such as improper disposal of carcasses of infected wild boars or infected pigs, disposed of by hunters and farmers. The sedentary nature of wild boars is considered the basic reason behind this issue. Generally, wild boars spend much time seated, up to 100 kilometers in 6 months; therefore, mostly they cannot cross long distances (67). Transmission of ASFV occurs during the migration of an adult male or female for reproduction purposes, the life span of the disease is short in this case ranging from five to 7 days (63). We assume that long-range ASFV incursions are not associated with wild boars; however, one cannot ignore their role in the transmission of ASFV to domestic pigs in close contact.

### Movement of virus through fomites

Recent experiments have suggested that the ASFV can remain in the blood, feces and urine of infected pigs. As ASFV can easily contaminate the environment, therefore, anything that is contaminated may act as a virus source (68). An example of this type of incidence occurred in Europe, where the disease was introduced by ships containing ASFV-contaminated kitchen and catering waste used to feed pigs near the surrounding areas. Subsequently, the disease spread to the Caucasian region, the countries of the European Union (69). However, fomites are usually considered equipment, clothing, bedding, footwear, or transport which is contaminated and whereby the virus can be moved to a new area (70). From the observation of these experimental procedures, the transfer and movements of ASFV with fomites should be considered as a possible way for ASFV to spread to virus-free areas.

### Anthropogenic factors

Human activities are important risk factors for ASFV transmission (26). Anthropogenic activities responsible for virus transmission include legal and illegal transport of pigs and pig’s products, insufficient biosecurity measures for pig holdings and noncompliance with hunting restrictions and control strategies during and after the ASFV outbreaks (71). The primary cause of ASFV transmission is anthropogenic activities that cause long-distance transmission events and the introduction of pig farms (72). In China an initial outbreak of ASFV was linked to the feeding of pigs with contaminated table scraps (73), and in Vietnam contaminated pork products were likely responsible for the first outbreak (74). In Asia, anthropogenic activities have played a key role in maintenance and transmission (74). Targeted interventions and advanced biosecurity measures are necessary to eradicate the transmission of ASFV due to human activities.

### African swine fever in Asia

In Asia, ASFV is more prevalent, following a pathway from northeast to southeast. In these countries, the transmission of ASFV is favored by compromised safety measures related to human activities including the transport of infected and contaminated fomites and pig products (75).

**Figure 2:**
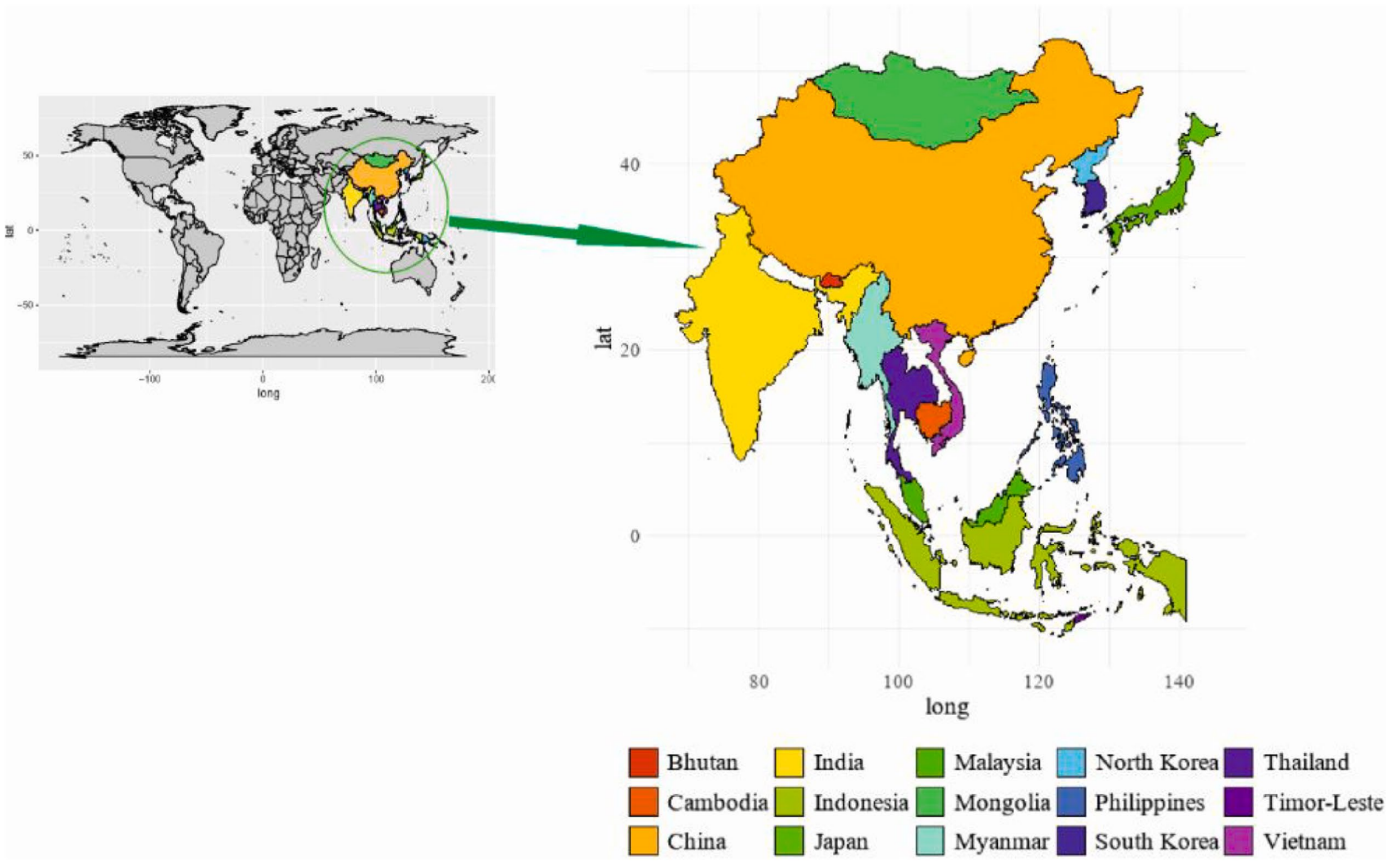
Geographical distribution of ASF in Asia.

The introduction of ASFV in Asia and especially the invasion of China had a drastic impact on the pig industry (76). Despite these efforts, the virus has persistently crossed international borders rapidly. Subsequent introductions have occurred in Cambodia, Bhutan, Malaysia, India, China, Indonesia, Mongolia, Thailand, North Korea, South Korea, Myanmar, Vietnam, East Russia, Timor Leste, the Philippines, and Hong Kong (Figure 2) (3). In 2019, ASFV outbreak reported in Mongolia was confirmed by the Organization for Animal Health or Office International des Epizooties (OIE) (77). Many of these countries are characterized by rural pig farms and small-holder operations, making the resulting outbreaks particularly challenging to monitor and manage effectively. The dynamic nature of the virus’s spread necessitates ongoing scientific efforts to understand and address the complexities of ASFV transmission within diverse agricultural and economic contexts (26).

### African swine fever in China

The rapid development of the Chinese economy has brought about significant changes in food consumption patterns. Similarly, demands for meat and meat products, especially pork, have shown a constant increase over the last few years (78). Taking this advantage, the swine sector of the country has integrated production systems to become an industry. Three types of pig farming have been practiced based on the number of pigs produced; small, medium and large farms. Farms close to large cities have been shifted from backyards to modern intensive farms (34).

In China, the first outbreak of ASFV was reported on August 3, 2018, when 400 pigs on a farm near Shenyang City in the north-eastern Liaoning province, developed acute clinical disease after consuming table scraps. The mortality rate was 100%, leading to abandonment of the farm. Subsequently, similar cases were observed on nearby farms (73). At the end of 2019, a total of 33 outbreaks were reported in eight provinces of the country, ASF has led to the deaths of more than 100,000 pigs with an estimated loss of USD 111.2 billion (79, 80). Without the availability of effective control measures, including vaccination, the resumption of production will be problematic. As a result, in China, pig production was reduced by 40% in 2019 compared to 2018. Similarly, the price of pork was doubled in 2019 (81). Therefore, the spread of this disease has posed threats to the large population of domestic pigs and wild boars in China, as well as in neighboring countries.

A strategy to control the outbreak has been developed by Chinese authorities (Ministry of Agriculture and Rural Affairs; MARA) and implemented soon after the emergence of the first outbreak. Culling all pigs within 3 km of the infected area and capturing all infected pigs, their disposals, and contaminants became mandatory. For the prevention and control of the ASFV outbreak, the Ministry of Agriculture and Rural Affairs has taken several preventive measures to control the outbreaks. Several preventive biosecurity measures were implemented including restricted pig movement, complete biosecurity protocols both inside and outside of pig farms, and systematic monitoring and recycling of pig products and waste materials. Quarantine measures have been enforced on farms, and high temperatures were applied for the treatment of feed and other waste materials of pigs (82). Despite these measurements, the epidemiological status in China become worse after the occurrence of ASFV new cases (83). The alarming spread and expansion of ASFV necessitates a dynamic scientific approach to control ASFV in various regions (84).

### African swine fever in Africa

**Figure 3:**
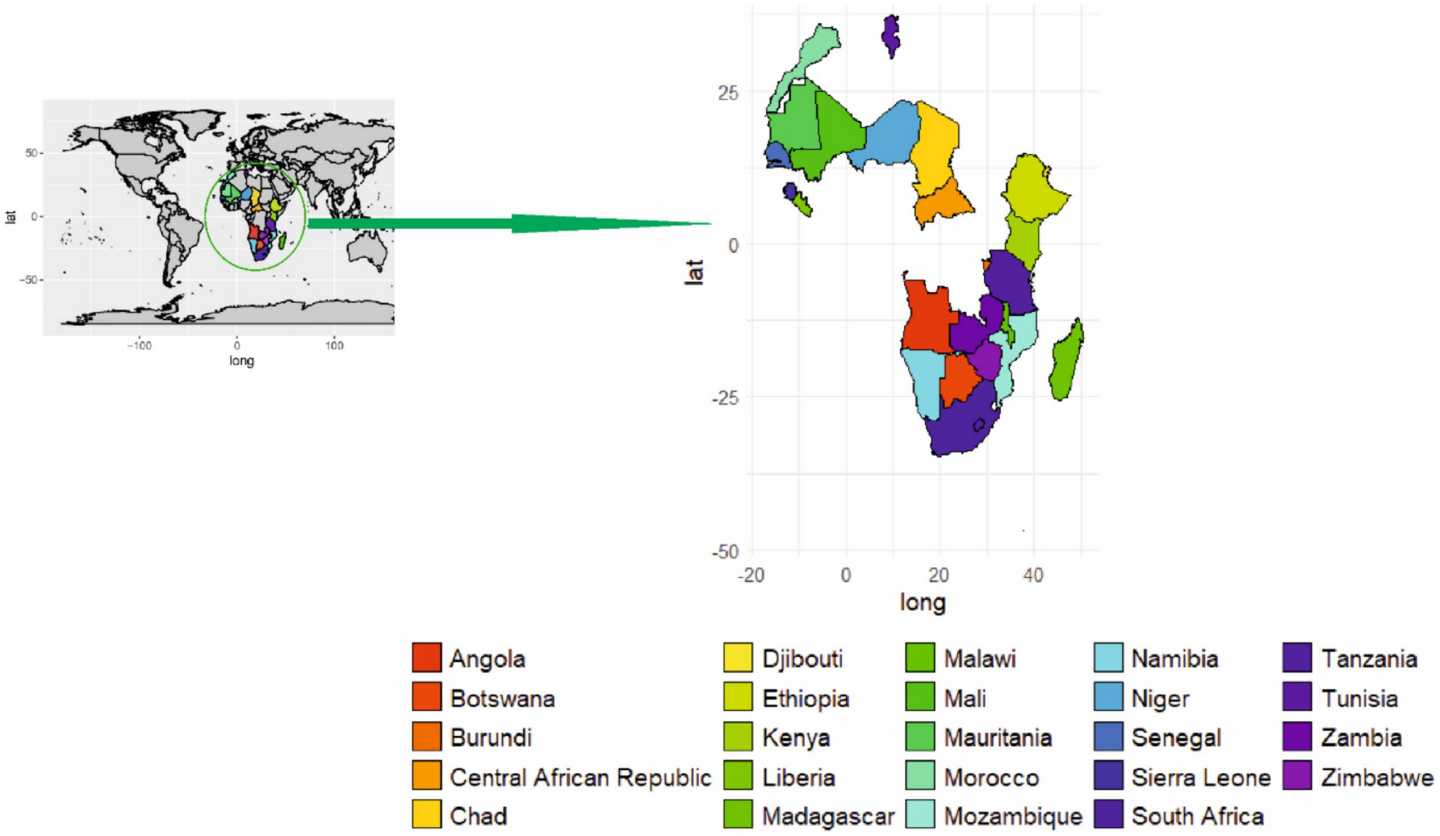
Geographical distribution of ASF in Africa.

In Africa, ASFV evolved through a sylvatic cycle between the soft tick *O. moubata* and warthogs (85). From 1989 to 2017, 5,134 ASFV outbreaks were recorded with 88.5% occurring in the domestic pig population and wild Suidae (33). The expansion and transmission of ASFV to new areas occurs due to the movement of infected pigs into ASFV free-areas, while warthogs and wild suids also play a major role in its transmission and propagation in Africa (9, 64). In Africa, the spread of ASFV to new areas beyond its endemic region causes severe losses (33). Outbreaks arise from transmission between domestic pigs and the sylvatic cycle, with warthogs being translocated into the southern part of the continent over the past four decades (7). In 2017, ASF outbreaks had been reported in Cabo Verde, Côte d’Ivoire, Burundi, the Central African Republic, Chad, Kenya, Madagascar, Mali, Mozambique, Namibia, Niger, Sierra Leone, South Africa, Zambia, Zimbabwe, Tanzania, Sahara, Zanzibar, Malawi, Angola, Botswana, Ethiopia, Djibouti, Liberia, Senegal, Mauritania, Morocco, Mali, and Tunisia [(Figure 3; (86)]. The rapid expansion of the African population has demanded an increase in meat production. This has a direct impact on the pig population, which constitutes approximately 5% of the global pig population (33, 87). Some African countries, namely South Africa, Madagascar, Mozambique, Namibia, and Nigeria, reported one to multiple outbreaks. Likewise, the unquantified presence of the disease has also been reported in countries like Cameroon and Cape Verde (33, 85). At the end of 2017, 69 cases of ASFV were confirmed in which warthogs act as a source of the outbreak that occurred in Kenya, Namibia, Botswana, Zambia, Zimbabwe, South Africa and Tanzania (33). Restriction of wild pigs to conservation areas has reduced their likely role in the epidemiology of ASFV (75).

### African swine fever in Europe

**Figure 4:**
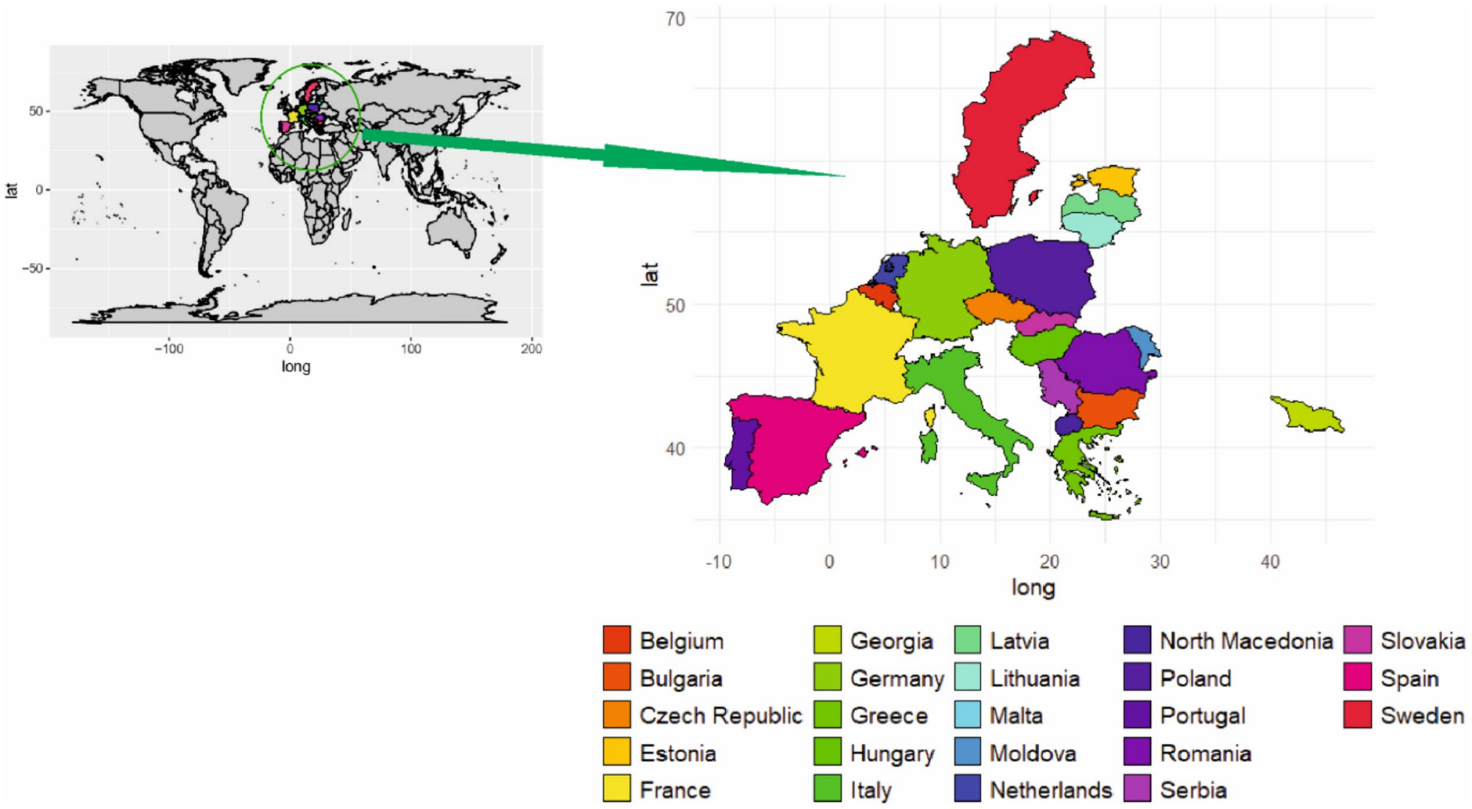
Geographical distribution of ASF in Europe.

In Europe, the past 6 years have seen the introduction of ASFV to Belgium, Bulgaria, the Czech Republic, France, Germany, Greece, Hungary, Italy, Lithuania, Malta, Moldova, the Netherlands, North Macedonia, Poland, Romania, Serbia, Slovakia, Spain, Sweden, Georgia, Portugal, Latvia, Estonia and Romania (Figure 4) (88). Recently, the Czech Republic and Belgium, in which domestic pigs were not infected, now appear to have eradicated ASFV via biosecurity measures. Elsewhere (including Bulgaria, Hungary, Poland, Romania, and Slovakia in particular), the virus generally appears to be beginning ground, with numerous outbreaks, especially on smallholder farms (86). Similarly, the introduction of ASFV in northeast Lithuania resulted in the death of more than 20,000 pigs (14). Epidemiological investigations have revealed some details of the ASFV transmission patterns unique to these countries (e.g., Poland, where wild boar infections are dominant, versus Romania, where domestic outbreaks are more common) and have also identified the apparent evolution of lower-virulence ASFV strains in Estonia and Latvia (89). The ASFV genotype I was restricted to the African continent from its first recognition in 1920 until 1957, when the outbreak was reported in Portugal. Spain was the first European country to report ASFV cases, followed by Italy, France, Malta, Belgium, and the Netherlands (28, 90). Pork meat imported directly from Spain was thought to be the source of the first ASFV case in Belgium in 1985. The first outbreak of ASF resulted in the slaughter of 34,000 pigs housed on 60 holdings (29). The pig population in the Netherlands was severely affected from 1960 to 1995 due to ASF (29, 91). Although ASF was successfully eradicated from most European countries by 1995, a notable exception was the Mediterranean island of Sardinia (Italy). The main factors that were responsible for the persistence of the disease in that area were the keeping of more than 70% of the pig population in extensive systems and backyard farms, combined with the proximity of wild boars (92).

The lack of consistency in ASF contingency plans and preventive measures resulted in the second spread of ASFV in European countries (29). In this context, the first case was recognized in Georgia in 2007,followed by numerous outbreaks of domestic pigs and wild boars (28, 29, 64, 90). The specific origin of the virus responsible for the outbreak is still unknown; however, the virus genotype has been linked with that in Madagascar and Mozambique (90). The disease spread from Georgia to native European countries and was recently reported in Latvia, Estonia, Hungary, and the Netherlands (90). Not only in western Europe, the pig population was also severely affected in eastern Europe. Poland, a country where 66% of the pig population is kept on small farms, reported the first case of ASF in 2014 (90, 93). Since the first outbreak in Poland, authorities have reported several outbreaks, with a total of 5,333 cases of the disease being confirmed in wild boars (29).

Since the first appearance of ASF in Europe in (1960), it took 30 years to successfully eradicate the virus from affected countries (94, 95). The second spread of the disease (2007), contributed to virus migration in nearby regions, as well as a high probability of outbreaks in neighboring countries (90). Efforts to control disease in Europe have not been successful. Impediments to the development of successful eradication programs include low biosecurity (human factor), free-ranging wild boar populations, and a high prevalence of the virus in surrounding bordering countries (90). Low or non-existent biosecurity measures at small-scale pig holdings increase the risk of introducing viruses on the farm. Ticks, wild boar populations, and the illegal trade of infected meat products are the other factors responsible for maintaining and circulating the virus within pig populations (57). Despite the challenges, the European authorities have implemented multifaceted preventive and hygiene procedures. In addition, government institutions are strongly convinced of achieving the eradication of disease in a short period of time.

### African swine fever in the United States and its future assessment

The United States (United States) is believed to manage pig production under high biosecurity conditions. Economic losses due to ASFV introduction into the U. S are estimated in between $15 and $50 billion, depending on the disease spread in the feral swine population (96). Commercial swine production is a closed system from farrowing to slaughter as a means of reducing the risk of pathogen introduction (97). To limit cross contamination, transport vehicles, animal feed, personnel and other fomites are closely managed. Despite the high-profile biosecurity measures, transport equipment contributed to the spread of the porcine epidemic diarrhea virus (PEDV) in 2013 (59). This indicates that despite stringent biosecurity protocols, it can be difficult to control ASF. Keeping this in mind, the US has substantially contributed to the implementation of a series of preventive measures designed for the importation of live animals and their products.

### Prevention and control strategies

The escalating intensification of animal movements and product exchanges have increased the risk of ASF in new regions, particularly those developing trade ties with new eastern EU member states (98). Despite of this threat, despite efforts, there is no effective vaccine available for global eradication. The developing of an effective vaccine becomes crucial to controlling one of the major pig diseases in Africa. Such a vaccine would offer an alternative to animal slaughter, mitigating the spread of ASF in both Africa and Europe (99). Challenges to eradication efforts include free-range production systems, interactions with *Ornithodoros* ticks and/or wild suids, and endemicity involving asymptomatic carriers (Wamwatila et al., 2015) (100). Success relies highly on effective communication between all involved parties in an outbreak, including diagnostic laboratories, farmers, field and official veterinarians, disease crisis centers, and media participation. Implementing improvements in pig housing to minimize contact with ticks and wild animals have proven highly efficient in reducing infection to eradication levels and should not be overlooked (1). A cost–benefit analysis should determine whether contingency efforts would be directed toward control or eradication (101).

In the absence of a vaccine and considering the role of the sylvatic cycle in the epidemiology of southern and eastern Africa’s disease, logical measures for ASF control include the physical separation of domestic pigs from wild hosts and treating pig premises with acaricides in areas where tick-infected by ASFV occurs (28). Control based on the physical separation between wildlife and pigs has been proven successful in controlling ASFV, even in animals from those regions where the virus circulates among infected warthog populations (7).

### Control strategy based on genetically modified vaccines

Despite the implementation of strong biosecurity measures, it is a difficult task to control the spread of disease in the pig population. Control measures rely on prompt reporting, repeat testing, and culling infected and at-risk animals. Moreover, in underdeveloped countries these methods and their establishment are difficult to apply. As an alternative, researchers are now struggling to develop an effective vaccine against ASFV. For this purpose, several attempts have been made in recent decades. Different vaccine strategies such as DNA vaccines, adenovirus vector vaccines, subunit vaccines, and inactivated vaccines have been tested and proven to be unsuccessful (2). Similarly, extracts of infected cells, purified and inactivated virions, infected leukocyte blood supernatants, infected glutaraldehyde fixed macrophages, or infected alveolar macrophages have been used to produce immunity against ASFV (62). However, all of these attempts failed to produce desirable results. Meanwhile, it was observed that pigs infected with attenuated or virulent variants of ASFV may establish resistance to homologous virus challenge (102). These observations led scientists to develop an effective live attenuated virus by deleting genes not associated with ASFV replication. Virulent isolates of ASFV have been modified with deletions of genes to attenuate the virus. Keeping this in mind, the BeninΔDP148R virus was genetically modified by deleting the *DP148R* gene to isolate the virulent strain, Benin97/1. Deletion of the gene reduced the pathogenicity of the BeninΔDP148R virus in pigs. All the pigs immunized with the virus showed only mild transient clinical signs and survived infection. Moreover, high level of protection was observed against the parental virulent strain (103). The same level of safety and protection was observed after immunization of the pig with ASFV-G-ΔI177L. After immunization, the pig showed a strong and specific antibody response and low viremia titers (104) (Table 2).

**Table 2:** Genetically modified ASFV by deletion of genes and their effects in immunized pigs.

Isolate	Gene deleted	Protection against parental virus	References
ASFV-G	TK (thymidine kinase)	No	(105)
ASFV-G (2007)	9GL (B119L)	High	(106)
NH/P68	A238L, A224L, EP153R and A276R	Moderate	(14)
Benin 97/1	DP148R	High protection	(103)
ASFV-G	I177L	High	(104)
ASFV-G (2007)	9GL and UK	High	(107)
HLJ/18	MGF505-1R, MGF505-2R, MGF505-3R, MGF360-12 L, MGF360-13 L, MGF360-14 L and CD2v.	High	(81)
Benin 97/1	MGF360 and MGF530/505	High	(103)
ASFV-G (2007)	MGF505-1R, MGF360-12 L, MGF360-13 L, MGF360-14 L, MGF505-2R, and MGF505-3R	High	(106)
OUR T88/3	DP71L and DP96R	Moderate	(106)
ASFV-G (2010)	8DR (EP402R)	Failed to induced	(104)
ASFV-G	9GL/NL/UK	Failed to induced	(104)
ASFV-G (2007)	9GL and MGF	Failed to induce	(106)

Based on available knowledge, the use of genetically modified viruses is the most reasonable approach to establishing an effective ASFV vaccine. Genetic modification and deletion of one or more genes change the virus from virulent to less virulent (104, 108). In domestic pigs ASAFV-G-Δ8DR is responsible for disease state. Pigs infected with the ancestral virus ASAFV-G-Δ8DR show the same viremia values. More attention is needed for the selection of targeted genes. Attenuated viruses of different genotypes should be tested to obtain strains that protect isolates circulating in different regions. Moreover, optimized targeted genes are used for safety standards. Similarly, the issue of the availability of a licensed cell line to grow live attenuated viruses for vaccine production needs to be resolved. For efficient control measures, immune responses induced by virus antigenic proteins are necessary to enhance the protection of infected animals (108). Therefore, the rational development of novel ASFV vaccines requires caution and more work to optimize commercial production.

## Conclusion

The current situation of ASF signifies a constant risk to the livestock sector. Recent exploration and flourishing of ASFV have demonstrated the ability of the virus to spread over long distances. As a result, there is a tremendous decrease in both the production and farming of pigs. Furthermore, the implications of the trade related to ASFV in swine have severely affected the pork industry. Veterinary services need to perform rigorous surveillance in countries that consume pigs, as the inaccessibility to effective medication persists, leading to high mortality rates are the main reasons. Biosecurity measures are crucial to prevent the transmission of viruses. Inadequate biosecurity practices can create opportunities for the spread of viruses, which pose risks to human and animal health. Vaccines have given some favorable results; however, further investigation is required to prove them as the only choice to treat and control the disease.

## Author contributions

BA: Formal analysis, Investigation, Methodology, Visualization, Writing – original draft, Writing – review & editing. C-HW: Writing – original draft, Writing – review & editing. MK: Writing – original draft, Writing – review & editing. MN: Writing – original draft, Writing – review & editing. C-CC: Writing – original draft, Writing – review & editing. AA: Conceptualization, Data curation, Formal analysis, Funding acquisition, Investigation, Methodology, Project administration, Resources, Software, Supervision, Validation, Visualization, Writing – original draft, Writing – review & editing.
